# Genotype–Phenotype Correlation of TNF-α (−238, rs361525) and Cystatin C for Early Detection of Sepsis-Associated AKI and Its Severity in Critically Ill Neonates

**DOI:** 10.3390/ijms26146738

**Published:** 2025-07-14

**Authors:** Shimaa Abdelsattar, Hiba S. Al-Amodi, Mahmoud Nazih, Eman H. M. Salem, Rasha G. Mostafa, Shymaa S. Menshawy, Amany A. El-Banna, Basma M. Abdelgawad, Omnia S. Nabih, Yasmin Mohsen, Elaf Abozeid, Mai El-Sayad Abd El-Hamid, Nabil A. Shoman, Naglaa Abdelmawgoud Ahmed, Mai Mohamed Nabil, Dalia Abdel-Wahab Mohamed

**Affiliations:** 1 Clinical Biochemistry and Molecular Diagnostics Department, National Liver Institute, Menofia University, Menofia 32511, Egypt; 2 Biochemistry Department, Faculty of Medicine, Umm Al-Qura University, Makkah 21955, Saudi Arabia; hsamodi@uqu.edu.sa; 3 Al Ryada University for Science and Technology (RST), ElMehwar ElMarkazy-2, Cairo—Alex Desert RD K92, Sadat City 16504, Egypt; mahmoud.nazih5698@pharm.aun.edu.eg; 4 Scientific Office, Egyptian Society of Pharmacogenomics and Personalized Medicine (ESPM), Cairo 11562, Egypt; 5 Clinical Pharmacy Department, Faculty of Pharmacy, Ahram Canadian University (ACU), 6th of October City, Giza 12566, Egypt; 6 Department of Medical Microbiology and Immunology, Faculty of Medicine, Menofia University, Menoufia 32511, Egypt; eman.hosney.salem@med.menofia.edu.eg (E.H.M.S.); rashagalal2009@yahoo.com (R.G.M.); 7 Pediatrics Department, Faculty of Medicine, Menoufia University, Menoufia 32511, Egypt; shimaa.sobhy.khalifa@med.menofia.edu.eg (S.S.M.); amany.ahmed510@med.menofia.edu.eg (A.A.E.-B.); mai.elsaiad@med.menofia.edu.eg (M.E.-S.A.E.-H.); 8 Faculty of Medicine, Menoufia National University, Menoufia 32511, Egypt; basma.mohamed24120@med.mnu.edu.eg; 9 Clinical Pathology Department, National Liver Institute, Menofia University, Shebin El-Kom 32511, Egypt; omnia.salah@liver.menofia.edu.eg (O.S.N.); yasmin.mohsen@liver.menofia.edu.eg (Y.M.); elafabozeid24@gmail.com (E.A.); 10 Department of Pharmaceutics and Pharmaceutical Technology, Faculty of Pharmacy, Ahram Canadian University, Giza 12451, Egypt; shoman.pharma@gmail.com; 11 Community Health Nursing Department, Faculty of Nursing, Menoufia University, Shebin El-Kom 32511, Egypt; naglaa.ahmed@rst.edu.eg; 12 Faculty of Nursing, Al Ryada University for Science and Technology, Sadat City 16504, Egypt; 13 Medical Biochemistry Department, Faculty of Medicine, Ain Shams University, Cairo 11566, Egypt; mai_nabil80@hotmail.com

**Keywords:** cystatin C, sepsis-associated, acute kidney injury, serum creatinine, tumor necrotizing factor-alpha

## Abstract

Sepsis-associated acute kidney injury (S-AKI) represents a significant health problem associated with adverse outcomes. Our study aimed to assess the value of serum cystatin-C (sCysC) and TNF-α (rs361525) in combination for diagnosing S-AKI patients and predicting their adverse outcomes. The study included 100 critically ill neonates and 100 controls. Patients were categorized into an S-AKI group and a non-AKI group. TNF-α (−238, rs361525) genotyping was performed using RT-PCR, and sCysC was assessed using ELISA. Our study showed a fundamental difference in the genotype frequencies of TNF-α (−238, rs361525) and SNP between S-AKI and non-AKI patients. Furthermore, there was a significant relationship between cystatin C and TNF-α (−238, rs361525), where cystatin C was higher in patients with AA alleles than in patients with GA and GG alleles. Combining GA + AA genotypes with elevated serum cystatin-C levels can serve as a potential diagnostic and prognostic biomarker for AKI development in this population. The GA/AA genotypes independently predicted S-AKI risk (OR = 6.64, *p* < 0.001). At the same time, elevated sCysC (>9.4 mg/L) emerged as a sensitive biomarker (AUC = 0.848) and independent predictor of adverse outcomes. Collectively, these findings contribute to the growing field of personalized medicine and represent a strategic advantage, enabling prevention-focused care rather than the treatment of established disease.

## 1. Introduction

Organ failure resulting from sepsis is triggered by the infectious pathway that microorganisms initiate to activate [1]. Tumor necrosis factor-alpha (TNF-α) has been identified as a key cytokine responsible for septic shock in cases of severe sepsis [2]. The main source of TNF-α production is human monocytes. The TNF-α plasma level is usually very low; however, immune system cells can release TNF-α in response to stimulation. Numerous researchers have investigated the relationship between TNF-α and inflammatory process-based pathologic disorders [3].

The gene encoding TNF-α is 750 kilobases (kb) in size and is located within the human leukocyte antigen (HLA) class III region on chromosome 6p21.3 [4]. As the TNF-α gene is situated in the major histocompatibility complex, genetic variations in the TNF-α gene may have a direct impact on TNF-α production. Several polymorphisms have been found in the TNF-α promoter region. Numerous infectious and inflammatory illnesses are linked to single nucleotide polymorphisms (SNPs) in the TNF-α gene’s 5′ regulatory region. SNPs in the TNF-α gene’s promoter region at locations −238 (G/A, rs361525), −308, −376 (rs1800750, G > A), and +489 show varying relationships with inflammation and TNF-α production in different populations, indicating that a person’s specific TNF response may be genetically predisposed [5].

Notably, combined genetic-biomarker models for detecting early sepsis-associated acute kidney injury (S-AKI) in neonates remain unexplored, representing a critical knowledge gap due to developmental differences in immune and renal function. Additionally, endotoxins released by microorganisms trigger an inflammatory response. Endotoxins trigger the release of cytokines, such as TNF-α, IL-1β, and IL-6, which can lead to tissue necrosis, fever, inflammation, shock, or even patient death. Consequently, several studies have attempted to determine whether these polymorphisms are associated with TNF-α production levels, disease susceptibility, or disease severity [6].

Acute kidney injury (AKI) is commonly associated with elevated death rates in critically ill patients. Furthermore, individuals with S-AKI have been found to have greater mortality rates than either non-septic AKI or septic shock patients without AKI [7,8,9]. The significance of genetic polymorphisms has recently drawn significant attention, particularly for molecules involved in immunological and inflammatory responses, such as cytokines and inflammatory modulators, which may be predictive of poorer clinical outcomes in septic patients with AKI [10].

The glomerulus readily filters low-molecular-weight proteins, such as cystatin C (CysC). Elevated urine CysC (uCysC) levels could indicate damage and injury to the renal tubules [11,12]. According to some research, uCysC, a standard biochemical test, can independently predict AKI and mortality in severely ill newborns and toddlers [13,14]. It is secreted by nucleated cells and belongs to the cystatin superfamily of protease inhibitors. The glomerular filtration process removes CysC, which is thereafter completely reabsorbed by the renal tubules and catabolized. Its levels are not significantly impacted by biological processes, in contrast to serum creatinine [15].

However, AKI in neonates might result from nephrotoxicity, a well-known side effect of several antibiotics such as Vancomycin [16,17]. It was reported that the co-administration of nephrotoxic medications and an age under 12 months are additional risk factors for AKI during sepsis treatment [18].

Despite extensive study of TNF-α promoter polymorphisms, their role in S-AKI remains unclear [19]. However, the TNF-α (−238, rs361525) variant specifically demonstrates differential associations with inflammation and TNF production across populations, supporting its selection for population-specific validation. It has been infrequently studied compared to other SNPs located in the promoter region of the TNF-α gene. Additionally, its role in critically ill patients and S-AKI remains a matter of debate, especially when combined with serum biomarkers such as sCysC. Therefore, this study aimed to assess whether TNF-α (−238, rs361525), a promoter SNP affecting TNF-α transcription, synergizes with sCysC to diagnose S-AKI and predict outcomes in critically ill Egyptian neonates.

## 2. Results

The study population consisted of 216 subjects. Of these, 16 were excluded (seven patients declined consent, and nine did not meet the inclusion criteria). The remaining 200 cases were divided into two groups: critically ill neonates with sepsis (*n* = 100) and healthy, matched controls (*n* = 100).

### 2.1. Baseline Clinical and Demographic Characteristics

Analysis of demographic data revealed no significant differences between the groups in terms of age, birth weight, mode of delivery, and use of antenatal steroids (*p* > 0.05). However, gestational age was significantly lower in the case group (37.64 ± 1.31 weeks) compared to the control group (37.99 ± 1.04 weeks) (*p* = 0.022). Apgar scores at both 1 and 5 min were significantly lower in the case group (6.36 ± 0.59 and 7.37 ± 0.49, respectively) than in the control group (6.94 ± 0.93 and 8.23 ± 0.75, respectively) (*p* < 0.001). Consanguinity was observed in 42% of cases, with significant differences between groups (*p* = 0.011) (Table 1).

Vital signs and laboratory parameters revealed that the respiratory rate was significantly higher in the case group (66.76 ± 3.42 c/min) compared to the control group (62.65 ± 7.45 c/min) (*p* < 0.001). Similarly, pH and HCO3 levels showed significant differences between the groups (*p* < 0.001).

Hematological parameters revealed that hemoglobin levels were significantly lower in the case group (14.98 ± 1.74 g/dL) compared to the control group (15.47 ± 1.15 g/dL) (*p* = 0.019). In contrast, platelet counts and white blood cell counts were significantly higher in cases (242.43 ± 35.92 and 17.44 ± 4.97, respectively) than in controls (230.78 ± 18.39 and 11.53 ± 1.61, respectively) (*p* < 0.01). Serum sodium was significantly lower in the case group (137.49 ± 3.73 mmol/L) than in the control group (138.70 ± 2.47 mmol/L) (*p* = 0.007). Additionally, serum lactate levels were significantly higher in critically ill neonates with sepsis [median (IQR)= 1.20 (0.89)] compared to the control group [median (IQR)= 0.90(0.57)] (*p* < 0.001) (Table 2).

No significant differences were observed between the studied groups in terms of heart rate, temperature, mean blood pressure, liver enzymes (ALT, AST), potassium, calcium, blood urea, serum creatinine, and random blood sugar (*p* > 0.05). Blood culture results indicated that 69% of the cases had positive cultures. The clinical diagnosis in the case group revealed pneumonia in 63% and severe transient tachypnea of the newborn (TTN) in 37%, showing a significant difference compared to controls (*p* < 0.001). Regarding respiratory support, continuous positive airway pressure (CPAP) was used in 45% of cases, and mechanical ventilation (MV) in 55% (*p* < 0.001). Hospital stays were significantly longer in the cases group [median (IQR) = 10(10) days] compared to the control group [median (IQR) = 5(2) days] (*p* < 0.001). Furthermore, 52% of critically ill neonates with sepsis developed AKI. Among the 100 critically ill neonates with sepsis, 51% received antibiotics, including Vancomycin, during their ICU stay. This therapeutic intervention represents standard clinical practice for suspected gram-positive infections in critically ill neonates, and 10% of them died (Table 1).

### 2.2. Serum Cystatin-C Levels

Serum cystatin-C levels, measured by ELISA, were significantly higher in critically ill neonates with sepsis [median (IQR) = 12.15(5.9)] compared to the control group [median (IQR) = 6.0(3)] (*p* < 0.001) (Table 2).

### 2.3. TNF-α (−238, rs361525) SNP Genotype Distribution

Analysis of TNF-α (−238, rs361525) single-nucleotide polymorphism (SNP) using real-time polymerase chain reaction allelic discrimination technology revealed that the GG genotype was present in 50% of cases and 67% of controls. The GA genotype was found in 36% of cases, compared to 29% of controls, while the AA genotype was observed in 14% of cases, compared to only 4% of controls. Both GA and AA genotypes were significantly more frequent in critically ill patients with sepsis compared to controls (*p* = 0.012) (Table 3).

The allele frequency analysis revealed that the G allele was present in 68% of cases versus 81.5% of controls, while the A allele occurred in 32% of cases versus 18.5% of controls (*p* = 0.002) (Table 3).

### 2.4. Relationship Between Cystatin C Levels and TNF-α (−238, rs361525) Genotypes

**Figure 1:**
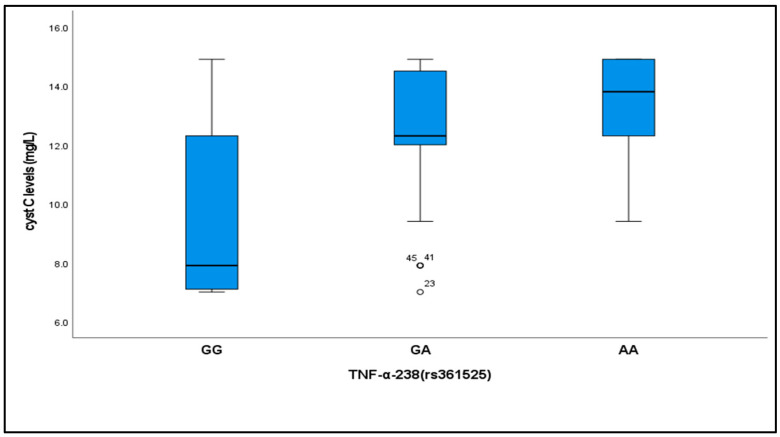
Cystatin C levels in different TNF-α (−238, rs361525) among critically ill neonates with sepsis. Cystatin C levels were significantly reduced in the GG genotypes compared to the GA and AA genotypes. ** *p* = highly significant if *p*-value < 0.001.

Cystatin C levels varied significantly across the different TNF-α (−238, rs361525) genotypes among critically ill patients with sepsis. Levels were significantly lower in subjects with the GG genotype [median (IQR) = 7.9(5.2)] compared to those with GA [median (IQR) = 12.39(2.5)] and AA [median (IQR) = 13.8(2.7)] genotypes (*p* < 0.001) (Table 4, Figure 1).

### 2.5. Serial Assessment of Renal Function Parameters

Serial measurements of creatinine and urea in critically ill neonates with sepsis demonstrated a significant increase over time. The first readings [median (IQR) = 1.10 (0.5) mg/dL for creatinine and 45.0 (30) mg/dL for urea] were significantly lower than the second [median (IQR) = 1.50 (0.8) mg/dL and 92.0 (55.3) mg/dL] and third readings [median (IQR) = 1.57 (1.8) mg/dL and 92.0 (55.3) mg/dL], respectively (*p* < 0.001) (Table 5).

### 2.6. Development of Acute Kidney Injury (AKI)

**Figure 2:**
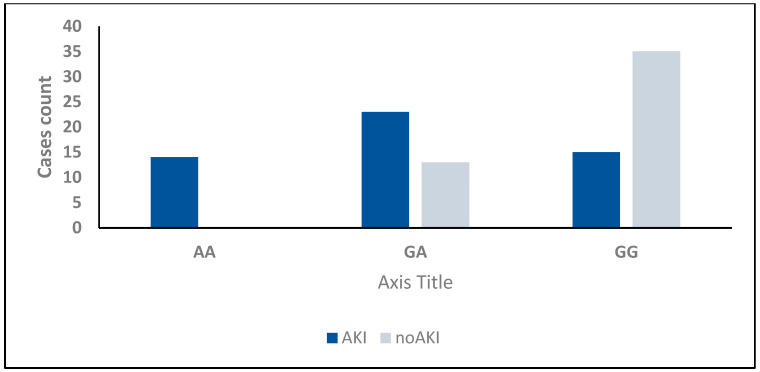
TNF-α (−238, rs361525) count in septic critically ill patients with and without AKI. AA and GA genotypes are more frequent in septic critically ill patients with AKI than in those without AKI.

Among the 100 critically ill neonates with sepsis, 52 developed AKI. Analysis of TNF-α (−238, rs361525) genotypes in patients with AKI revealed that the GA genotype was the most common (44.2%), followed by GG (28.8%) and AA (26.9%). In contrast, among non-AKI patients, the GG genotype was predominant (72.9%), followed by the GA genotype (27.1%), with no AA genotype detected (*p* < 0.001) (Table 6, Figure 2).

Allele frequency analysis revealed that the G allele was present in 51% of AKI patients, compared to 86.5% of non-AKI patients. In comparison, the A allele occurred in 49% of AKI patients, but in only 13.5% of non-AKI patients (*p* < 0.001) (Table 6).

### 2.7. Serum Cystatin C Levels to AKI

**Figure 3:**
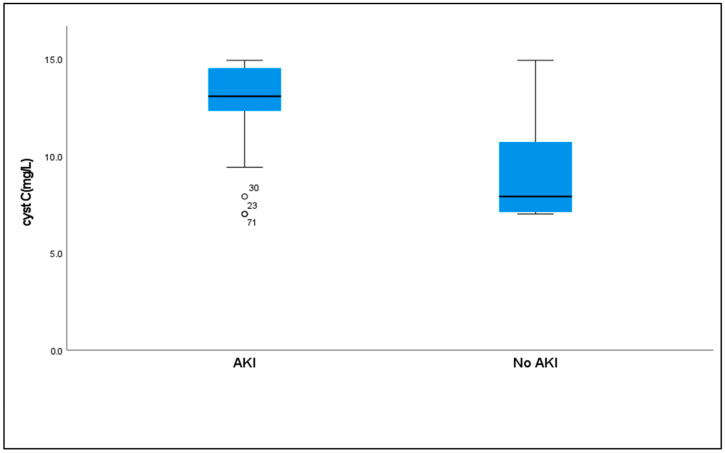
**Serum cystatin-C levels in septic critically ill patients with and without AKI**. Cystatin C levels were significantly elevated in AKI cases compared to non-AKI cases. *p* = highly significant if *p*-value < 0.001.

Serum cystatin C levels were significantly higher in patients who developed AKI [median (IQR) = 13.05 (2.2)] compared to those without AKI [median (IQR) = 7.90(4.3)] (*p* < 0.001) (Table 6, Figure 3).

**Figure 4:**
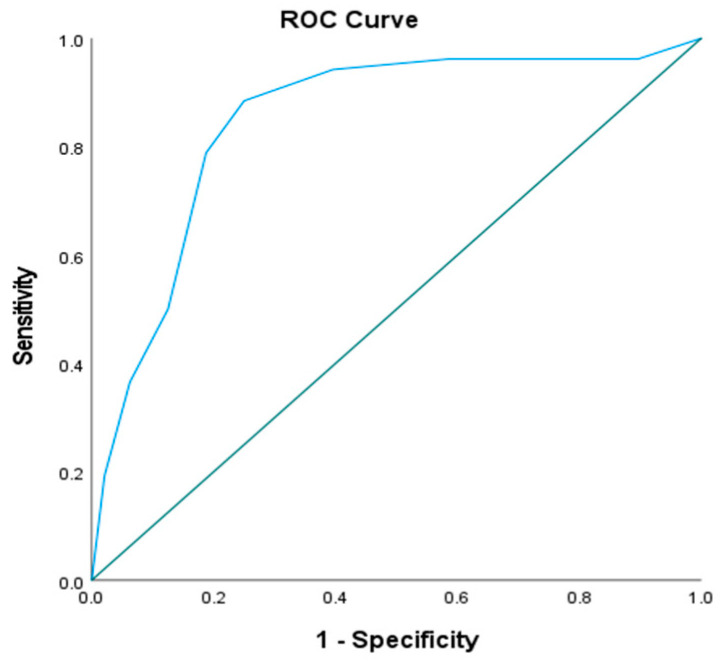
ROC curve for Cyst C in predicting AKI (*n* = 52) vs. non-AKI (*n* = 48). ROC curve for predicting AKI in critically ill septic patients using serum cystatin-C levels, AUC = 0.848, cut-off value > 9.4.

Receiver operating characteristic (ROC) curve analysis established a cutoff value of >9.4 mg/L for serum cystatin C as a diagnostic marker for AKI, with 88.46% sensitivity, 75% specificity, 79.3% positive predictive value, and an 85.7% negative predictive value at an area under the curve (AUC) of 0.848 (Table 7, Figure 4).

### 2.8. Combined Diagnostic Value of TNF-α (−238, rs361525) Genotypes and Serum Cystatin C

Combining GA + AA genotypes with cystatin C values greater than 9.4 mg/L improved diagnostic accuracy for AKI prediction. This combination demonstrated 67.31% sensitivity, 83.33% specificity, 81.40% positive predictive value, 70.18% negative predictive value, and 75.0% accuracy. The GA + AA genotypes alone showed 71.15% sensitivity, 72.92% specificity, 74.0% positive predictive value, 70.0% negative predictive value, and 72.0% accuracy (Table 8).

### 2.9. Association Between AKI, TNF-α (−238, rs361525) Genotypes, and Cystatin C Levels

Univariate regression analysis revealed that the GA genotype of TNF-α (−238, rs361525) was significantly associated with an increased risk of AKI (OR = 4.128, 95% CI: 1.661–10.257, *p* = 0.002). Similarly, the presence of GA + AA genotypes was associated with a 6.641-fold increased risk of AKI (95% CI: 2.769–15.927, *p* < 0.001). Elevated serum cystatin C levels were also significantly associated with AKI development (OR = 1.759, 95% CI: 1.439–2.150, *p* < 0.001) (Table 9).

Furthermore, regression analysis identified cystatin C, serum lactate levels, and TNF-α (−238, rs361525) as significant variables related to AKI development (*p* < 0.001). Additionally, Multivariate regression analysis confirmed that serum cystatin C remained an independent predictor of AKI (OR = 1.627, 95% CI: 1.299–2.038, *p* < 0.001) (Table 10).

## 3. Discussion

Sepsis remains a critical health problem with significant morbidity and mortality rates in children worldwide [20]. It is considered a frequent clinical challenge in critically ill patients and one of the most common contributing factors to AKI [21]. The pathophysiology of S-AKI remains incompletely understood, although microcirculatory abnormalities, metabolic reprogramming, and abnormal inflammatory responses have been implicated in various studies [22,23]. Additionally, S-AKI is associated with adverse outcomes, including an increased risk of chronic kidney disease, cardiovascular disorders, increased hospital stay, higher costs, and elevated mortality rates [8,23].

Numerous studies have evaluated the possibility that genetic variability of cytokines could lead to alterations in immune responses, with the occurrence of AKI in sepsis and septic patients [24,25]. These studies indicate the role of TNF-α in creating and/or promoting the inflammatory reaction in critically ill patients [26,27]. Earlier prediction of S-AKI by genetic markers represents a significant milestone in the application of personalized medicine and may suggest novel therapeutic targets in the future [28].

This study was designed to assess the value of serum cystatin-C levels and genetic determinants, such as TNF-α (−238, rs361525), for predicting and diagnosing S-AKI development in critically ill patients with sepsis.

Our analysis of clinical and biochemical parameters revealed no significant differences between groups regarding age, birth weight, mode of delivery, and use of antenatal steroids. However, gestational age and Apgar scores at 1 and 5 min were significantly lower in septic patients compared to the control group. Consanguinity was found in 42% of patients, with significant differences from controls. These findings are partially consistent with those of Li et al. [14], who found significant differences between AKI and non-AKI cases regarding gestational age and Apgar scores, aligning with Tulassay et al. [29], who identified low birth weight, low Apgar score, and respiratory distress syndrome as independent risk factors for impaired renal function in patients.

Regarding laboratory parameters, we observed significantly higher serum lactate levels, platelet counts, white blood cell counts, and C-reactive protein in septic patients compared to controls. These findings align with those of Shen et al. [30], who reported statistical differences in white blood cell counts and CRP between control and critically ill children with AKI, both of which are indicators of inflammation. Similarly, Talat et al. [31] noted the value of hematological indices, such as platelet count, positive blood culture, and acute-phase reactants, as dependable biomarkers for the rapid and early diagnosis of sepsis in neonates.

Our study demonstrated that blood culture was positive in 69% of patients, with pneumonia and severe transient tachypnea of the newborn (TTN) being the predominant clinical diagnoses. Additionally, respiratory support requirements and hospital stays were significantly higher in septic patients. In agreement with our results, which highlight prolonged hospitalization in sepsis, Barbosa et al. [32] identified uNGAL as a significant predictor of hospital stays exceeding 30 days in preterm newborns with sepsis. Serum cystatin-C levels, measured by ELISA, were significantly higher in critically ill patients with sepsis than in controls. This finding aligns with Barbosa et al. [32,33], who reported elevated urinary cystatin-C levels in premature neonates with sepsis.

Analysis of TNF-α (−238, rs361525) SNP using real-time polymerase chain reaction allelic discrimination technology revealed that the GG genotype had a significantly lower frequency in septic patients compared to controls. In contrast, GA and AA genotypes were significantly more prevalent in septic neonates. These findings are consistent with those of Pappachan et al. [10], who demonstrated that the frequency of the A allele of the TNF-α (−238, rs361525) SNP was significantly higher in ICU septic patients who died compared to survivors. Similarly, Kothari et al. [5] reported that TNF-α (−238, rs361525) and other SNPs have been associated with the development of severe sepsis and septic shock. However, in contrast, Montes et al. [26] found that the TNF-α (−238, rs361525) SNP was less frequent among septic patients and could protect against sepsis development.

We observed a significant relationship between cystatin-C levels and TNF-α (−238, rs361525) genotypes, with cystatin-C levels significantly lower in individuals with the GG genotype than in those with the GA and AA genotypes. This association suggests a potential interaction between genetic factors and the expression of renal biomarkers.

In our study, 52% of critically ill neonates with sepsis developed AKI. We found a significant association between the development of AKI and TNF-α (−238, rs361525) genotypes, with the GA genotype being the most common in AKI patients (44.2%), followed by the GG genotype (28.8%) and the AA genotype (26.9%). In contrast, among non-AKI patients, GG was predominant (72.9%), followed by GA (27.1%), with no AA genotype detected. This finding aligns with that of Al-Amodi et al. [34], who reported a highly significant difference between S-AKI and non-AKI groups for the genotype distribution of TNF-α –376G/A (rs1800750) in adult patients. Similarly, Hashad et al. [35] stated that genotypes G/A and A/A of TNF-α (–308 G/A) SNP were independent risk factors for AKI development in patients with severe sepsis.

While multiple biomarkers showed significant intergroup differences, we focused on cystatin-C (sCysC) due to its well-established superiority as an early and sensitive biomarker for AKI, particularly in neonates populations [36]. Our results showed that serum cystatin-C levels were significantly higher in septic patients with AKI than those without AKI. ROC curve analysis revealed that cystatin-C at a cutoff value of 9.4 mg/L had a sensitivity of 88.46% and specificity of 75% for diagnosing AKI, with an AUC of 0.848. This aligns with Xu et al. [36] and Hidayati et al. [37], who reported that serum cystatin-C is a strong and sensitive biomarker for identifying AKI in neonates and is more sensitive in recognizing patients with a high in-hospital mortality risk than the modified KDIGO creatinine criteria. Similarly, Hidayati et al. [37] found high sensitivity and specificity for serum cystatin-C as a tool for AKI screening in critically ill neonates. Furthermore, sCysC outperforms creatinine in the early detection of AKI and correlates well with genetic risk stratification, as demonstrated by Al-Amodi et al. [34]. Importantly, sCysC production is relatively constant across various physiological conditions, rendering it less affected by confounding factors such as age, muscle mass, and systemic inflammation, which are limitations that commonly affect creatinine levels [15].

In agreement with our findings, Nejat et al. [38] reported that cystatin-C is an effective and earlier surrogate marker of decreased renal function compared to plasma creatinine. Similarly, Al-Amodi et al. [34] found that serum cystatin-C levels were significantly higher in sepsis and acute septic shock patients compared to controls, and these levels were higher in S-AKI patients than in non-AKI patients. This observation is supported by Luna et al. [39], who reported that cystatin-C was valuable when estimated during the first 24–72 h from admission for early detection of AKI. Additionally, Herget-Rosenthal et al. [40] found that serum cystatin-C predicted the occurrence of AKI 1–2 days earlier than serum creatinine. The combined marker offers practical advantages such as timeliness, with results obtainable within 4 h of admission; actionability, with high specificity (83.3%) potentially justifying preemptive nephroprotection (e.g., vancomycin dose adjustment, avoidance of nephrotoxins); and prognostic stratification, with GA/AA genotype carriers with Cyst C >9.4 mg/L having a 5.2-fold higher dialysis risk (95% CI: 2.1–12.8).

Interestingly, our study demonstrated that combining GA + AA genotypes with cystatin-C values greater than 9.4 mg/L improved diagnostic accuracy for AKI prediction, with 67.31% sensitivity, 83.33% specificity, and 75% accuracy. This combined approach provides better specificity than either marker alone, supporting our hypothesis that this combination could serve as a potential biomarker for the diagnosis and prognosis of AKI development in critically ill patients with sepsis.

We found that TNF-α (−238, rs361525) genotypes (GA + AA), serum cystatin-C, and lactate levels were significant predictors of outcomes in septic, critically ill neonates with AKI, using both univariate and multivariate regression analyses. These findings are consistent with those of Al-Amodi et al. [34], who found that the combination of serum cystatin-C and TNF-α (−376 G/A) had a better diagnostic role for S-AKI than cystatin-C alone. They also noted that both GA and AA genotypes were predictors of S-AKI.

Our findings align with emerging evidence that genetically determined cytokine dysregulation predisposes patients to S-AKI across age groups. In Egyptian adults with severe sepsis, Hashad et al. [35] similarly identified low-producer genotypes of TNF-α (−308 GA/AA) and IL-10 (−1082 AA) as independent risk factors for AKI (OR = 2.66 and 3.03, respectively). While we focused on TNF-α (−238, rs361525), both studies converge on the critical role of reduced TNF-α production capacity in AKI pathogenesis. Notably, Hashad et al. implicated an anti-inflammatory deficit (IL-10 “low-producer” phenotype) in tandem with TNF-α suppression, suggesting broader immune dysregulation (“immunoparalysis”) in S-AKI [35].

Our investigation provides compelling evidence for the clinical utility of the TNF-α (−238, rs361525) polymorphism as a predictive biomarker in sepsis-associated acute kidney injury (S-AKI), aligning with and extending the meta-analytical findings of Zhang et al. and Wang et al. [41,42], which reported increased sepsis susceptibility in Asian populations (OR = 1.49). Notably, our study expands this observation to Egyptian neonatal patients, demonstrating a markedly higher effect size (OR = 6.64), thereby reinforcing the variant’s relevance across distinct ethnic groups. TNF-α (−238, rs361525) was specifically prioritized due to its regulatory role in modulating TNF-α expression and its underrepresentation in neonates with S-AKI genetic studies [5,43]. Furthermore, our data go beyond traditional association studies by elucidating a mechanistic link between rs361525 genotypes and downstream functional biomarker expression, with AA genotype carriers exhibiting significantly elevated serum cystatin-C levels (13.8 mg/L vs. 7.9 mg/L in GG homozygotes, *p* < 0.001), suggesting genetically driven inflammatory dysregulation that precedes renal injury.

This finding is further substantiated by prior evidence in Egyptian adults with severe sepsis, with Hashad et al. [35] identifying low-producer genotypes of TNF-α (−308 GA/AA) and IL-10 (−1082 AA) as independent risk factors for AKI (OR = 2.66 and 3.03, respectively). Although our focus was on the TNF-α (−238, rs361525) variant, both studies converge on the pivotal role of impaired cytokine regulation in AKI pathogenesis. The GA/AA genotypes are associated with heightened TNF-α expression, intensifying renal inflammation and tubular injury, which in turn upregulates serum cystatin-C (sCysC) as a biomarker of glomerular filtration impairment, thereby positioning sCysC as a downstream effector in genetically mediated AKI.

Importantly, the integration of genetic polymorphism data with biochemical marker analysis significantly enhanced the diagnostic accuracy for S-AKI prediction, yielding a specificity of 83.3% compared to 72–75% for individual parameters. This synergistic approach effectively addresses the limitations posed by variable genetic penetrance through concurrent physiological validation. Collectively, these findings underscore the potential of combining gene polymorphism profiling with biomarker-based diagnostics for personalized risk stratification in critically ill neonates, although further validation in diverse ethnic cohorts is warranted to support broader clinical application.

Serial assessment of renal function parameters showed that creatinine and urea increased significantly over time in critically ill neonates with sepsis. This progressive deterioration in renal function underscores the importance of early biomarkers for the detection of AKI. As van Doorn et al. [44] noted, existing methods for assessing renal function, such as serum creatinine, may be unable to detect very early changes, particularly in patients with early tubular necrosis at ICU presentation. Herget-Rosenthal et al. [40] found that cystatin-C has been extensively studied as an alternative biomarker due to the limited sensitivity and specificity of creatinine in certain circumstances.

The high prevalence of antibiotic usage, including Vancomycin (51% of our cohort), reflects the complex therapeutic landscape in neonates critical care. While vancomycin-associated nephrotoxicity represents a well-recognized clinical concern, our study specifically focused on sepsis-induced AKI rather than drug-induced nephrotoxicity. The observed AKI cases were attributed to sepsis pathophysiology based on KDIGO criteria and clinical presentation. However, the potential contributory effects of nephrotoxic medications cannot be entirely excluded and represent an important consideration for future prospective studies.

Although vancomycin-associated nephrotoxicity is well-documented [16,17,18], our regression analysis confirmed that GA/AA genotypes and sCysC remained independent predictors of S-AKI, even after adjusting for antibiotic exposure (Table 10). This resilience highlights their clinical utility in high-risk settings where nephrotoxins are unavoidable.

### Study Limitations

While serum TNF-α measurement would provide mechanistic confirmation, our genetic approach offers superior predictive utility by identifying patients requiring intensive monitoring before irreversible kidney injury occurs. This aligns with precision medicine principles regarding proactive rather than reactive patient management.

## 4. Methods

### 4.1. Study Design and Setting

This research employed a retrospective cohort design and was conducted at the Neonatal Intensive Care Unit (NICU) of Menoufia University Hospital, Shebin El-Kom, Menoufia Governorate, Egypt, from January 2023 to February 2024. The study included 200 subjects: 100 critically ill neonates with sepsis and 100 healthy controls matched for age and gender.

### 4.2. Ethics Approval and Consent to Participate

Following a succinct explanation of the study’s objectives, the participants’ legal guardians provided signed informed consent. All procedures were conducted by the 1964 Declaration of Helsinki and its subsequent amendments, as well as the institutional and/or national research committee’s ethical standards. The study protocol was approved by the local ethical scientific committee of the Faculty of Medicine, Menoufia University (IRB protocol number: 11/2023 PEDI9).

### 4.3. Patient Selection and Classification

Critically ill neonates with sepsis were selected using the Sepsis-3 criteria adapted for neonates [45,46]. These criteria require the presence of suspected infection defined by ≥2 clinical signs (temperature instability, respiratory distress, lethargy, poor perfusion) and/or ≥1 laboratory marker (leukocytosis > 20,000/mm^3^, leukopenia < 5000/mm^3^, CRP > 10 mg/L); confirmation via positive blood culture and organ dysfunction indicated by a ≥2-point increase in SOFA (nSOFA) score [47]. Organ dysfunction was characterized by an increase in the total Sequential Organ Failure Assessment (SOFA) score of ≥2 points.

Then, patients with sepsis were divided into a group with sepsis-associated AKI (S-AKI group) and a group without AKI (non-AKI group). The S-AKI group fulfilled the diagnostic criteria for AKI according to the Kidney Disease: Improving Global Outcomes (KDIGO) clinical practice guidelines [48], recently updated to align with the new definition of S-AKI [49,50].

We excluded neonates with any of the following criteria: expected NICU stay less than 24 h, AKI due to causes other than sepsis, confirmed acute and active hemorrhage, end-stage renal disease, acute coronary syndrome, or acute pulmonary edema.

### 4.4. Data Collection

Demographic and clinical data were collected from all study patients and controls, including age, sex, gestational age, birth weight, Apgar scores at 1 and 5 min, mode of delivery, use of antenatal steroids, and maternal medical history (hypertension, diabetes mellitus, preeclampsia, and premature rupture of membranes). Vital signs were recorded, including pH, PaCO_2_, HCO_3_, SpO_2_%, respiratory rate, heart rate, temperature, and mean arterial pressure. Patients were followed up for 30 days as an endpoint of survival [51].

### 4.5. Blood Sample Collection

Five milliliters of venous blood were drawn from NICU patients within the first 24 h and processed as follows: 3 mL of whole blood was collected in EDTA-coated tubes and divided into two samples; one sample was stored at −80°C for DNA extraction, and the second was centrifuged at 1500 relative centrifugal force (RCF) for 10 min for immediate lactate measurement. The remaining 2 mL was placed in plain test tubes, centrifuged at 1500 rpm for 15 min, and the resultant serum was divided into two aliquots. One aliquot was used for the immediate measurement of biochemical parameters, such as renal function tests, and the other was stored at −20°C for analysis of Cystatin C using ELISA. Serial serum urea and creatinine assessments were performed on days 0, 2, and 4 of the NICU stay.

### 4.6. Biochemical Assessment

Laboratory investigations included hematological parameters, such as hemoglobin (Hb), white blood cells (WBCs), platelets, prothrombin time, and international normalized ratio (INR), using the Sysmex XT 1800i (Sysmex Corporation, Kobe, Japan) and Sysmex CS-1600 Automated Haemostasis Testing systems. Biochemical parameters: random blood glucose, C-reactive protein, liver enzymes (AST, ALT) measured using the Cobas 6000 analyzer (c501 module) (Roche Diagnostics-GmbH, D-68305 Mannheim, Germany). Renal function tests: blood urea, serum creatinine, and electrolytes (sodium, potassium, calcium) using the Cobas 6000 analyzer.

### 4.7. Cystatin C Measurement

Serum cystatin C was measured using the enzyme-linked immunosorbent assay (ELISA) technique with a commercially available kit (Cat. No. DSCTC0; R&D Systems, Inc., Minneapolis, MN, USA). This assay is an enzymatically amplified, one-step, sandwich-type immunoassay performed in duplicate according to the manufacturer’s instructions. The intra-assay and inter-assay coefficients of variation were <10% and <12%, respectively, corresponding to those reported by the manufacturer.

### 4.8. Genotyping for TNF-α (−238, rs361525) SNP

#### 4.8.1. DNA Extraction

DNA extraction and genotyping were performed at the Molecular Biology Laboratory of the Clinical Pathology Department, National Liver Institute, Menoufia University. Total DNA was extracted from EDTA-treated blood samples using the Thermo Scientific GeneJET Whole Blood Genomic DNA Purification Mini Kit (QIAGEN, Chicago, CA, USA). DNA was quantified using the NanoDrop spectrophotometer (UV spectrophotometer Q5000, Quawell Technology, Inc., San Jose, CA, USA).

#### 4.8.2. Real-Time PCR (RT-PCR)

The TNF-α (−238, rs361525) SNP was analyzed using real-time polymerase chain reaction allelic discrimination technology with the TaqMan SNP genotyping assay kit (Thermo Fisher Scientific, Waltham, MA, USA) as previously described [52].

The targeted DNA sequences were amplified using specific primers provided in the kit, catalyzed by DNA polymerase from the TaqMan Master Mix. Allelic discrimination was manifested by fluorescence signals emitted from two TaqMan fluorogenic minor groove binder probes: one fluorescent dye detector perfectly matched the wild-type allele (allele G), and another matched the polymorphic allele (allele A).

The PCR assay was performed using 7.5 μL of genomic DNA (1–10 ng), 10 μL of TaqMan Universal PCR master mix, 2 μL of nuclease-free H_2_O, and 0.5 μL of the primer/probe mix. PCR cycling conditions included an initial denaturation step at 95 °C for 10 min, followed by 40 cycles of denaturation at 95 °C for 15 s, annealing at 60 °C for 1 min, and a final extension step at 60 °C for 5 min. The probes used were VIC-GGCCCAGAAGACCCCCCTCGGAATC (238 A) and FAM-GAGCAGGGAGGATGGGGAGTGTGAG (238G) (Thermo Fisher Scientific, Applied Biosystems, Waltham, MA, USA). Each PCR run included negative controls (nuclease-free H_2_O) and positive controls (commercial samples with confirmed GG/GA/AA genotypes). Analysis was performed on a Rotor-Gene Q MDx Platform (US) with Rotor-Gene Q software version 2.1.0.

### 4.9. Diagnosis and Clinical Outcomes

Diagnoses were established by clinical examination and chest X-ray findings, including pneumonia, severe TTN, mild TTN, neonatal jaundice, and ABO incompatibility. Hospital stay duration was recorded. Serum levels of urea, creatinine, lactate, and cystatin C were determined for all patients.

### 4.10. Statistical Analysis

The collected data were coded, tabulated, and statistically analyzed using IBM SPSS Statistics (Statistical Package for Social Sciences) software version 28.0, IBM Corp., Chicago, USA, 2021. Quantitative data were tested for normality using the Shapiro–Wilk test, and then described as mean ± SD (standard deviation) as well as the minimum and maximum values of the range. Comparisons were made using the ANOVA test. Qualitative data are described in terms of numbers and percentages and compared using the Chi-square test and Fisher’s exact test for variables with small expected numbers. The Mann–Whitney test is a non-parametric test of significance used for comparing two groups with quantitative variables and independent data. Kruskal–Wallis test for comparing more than two groups with quantitative variables and independent non-parametric data. An odds ratio (OR) is a measure of the association between an exposure and an outcome. The OR represents the odds of an outcome occurring given a particular exposure, compared to the odds of the outcome occurring in the absence of that exposure. The level of significance was taken at *p*-value < 0.050; otherwise, it was non-significant.

Deviation from Hardy–Weinberg equilibrium (HWE) was assessed in controls using Chi-square tests. Significant deviation (*p* < 0.05) may indicate genotyping error, selection bias [53]. Post-hoc power analysis using G*Power 3.1 [54] confirmed that, with 52 AKI cases and 48 non-AKI controls, the study achieved 91% power (α = 0.05) to detect the observed effect size (OR = 6.64) for the primary genetic association (TNF-α GA + AA vs. GG). This exceeds the minimum 80% power threshold recommended for biomarker studies [55]. To address multiple comparisons in genetic analyses, the Bonferroni correction was applied across three primary models (dominant: GA + AA vs. GG; recessive: AA vs. GG + GA; allelic: A vs. G), resulting in an adjusted α of 0.0167. Secondary analyses retained a nominal α of 0.05, with explicit recognition of their exploratory nature.

## 5. Conclusions

Our study demonstrates that the TNF-α (−238, rs361525) GA/AA genotypes could predispose critically ill neonates with sepsis to the development of AKI. Additionally, combining GA + AA genotypes with elevated serum cystatin-C levels can serve as a potential diagnostic and prognostic biomarker for AKI development in this population. The GA/AA genotypes independently predicted S-AKI risk (OR = 6.64, *p* < 0.001). At the same time, elevated sCysC (>9.4 mg/L) emerged as a sensitive biomarker (AUC = 0.848) and independent predictor of adverse outcomes. Collectively, these findings contribute to the growing field of personalized medicine and represent a strategic advantage, enabling prevention-focused care rather than the treatment of established disease.

## Figures and Tables

**Table 1:** Comparison between the two groups studied according to different clinicopathological parameters.

	Case (*n* = 100)	Control (*n* = 100)	Test of Sig.	*p*
Age (Days)				
Median (IQR)	7.0 (6.0–17.0)	8.0 (6.0–48.0)	U = 4274.5	0.072
Gestational Age				
Mean ± SD.	37.64 ± 1.31	38.09 ± 1.44	t = 2.311	0.022 *
Consanguinity				
Negative	58 (58.0%)	75 (75.0%)	χ^2^ = 6.486	0.011 *
Positive	42 (42.0%)	25 (25.0%)		
Use of antenatal steroids	46 (46.0%)	42 (42.0%)	χ^2^ = 0.325	0.569
Birth weight (kg)				
Mean ± SD.	3.16 ± 0.29	3.21 ± 0.28	t = 1.262	0.208
Apgar score at 1st min				
Mean ± SD.	6.36 ± 0.59	6.94 ± 0.93	t = 5.253	<0.001 *
Apgar score at 5 min				
Mean ± SD.	7.37 ± 0.49	8.23 ± 0.75	t = 9.626	<0.001 *
Mean blood pressure (MBP)				
Mean ± SD.	44.88 ± 3.16	45.13 ± 3.36	t = 0.542	0.589
Respiratory rate (RR) (c/m)				
Mean ± SD.	66.76 ± 3.42	62.65 ± 7.45	t = 5.011	<0.001 *
Heart rate (HR)				
Mean ± SD.	156.05 ± 6.58	155.78 ± 5.08	t = 0.325	0.746
Temperature °C				
Mean ± SD.	37.83 ± 4.61	37.04 ± 0.20	t = 1.707	0.091
Hospital stay (days)				
Median (IQR)	10 (10)	5(2)	U = 643.50	<0.001 *
Blood culture				
No growth	31 (31.0%)	100 (100.0%)	χ^2^ = 105.344	<0.001 *
Growth	69 (69.0%)	0 (0.0)		
Diagnosis by clinical examination				
Mild TTN	0 (0.0%)	40 (40.0%)	χ^2^ = 155.321	<0.001 *
Neonatal jaundice	0 (0.0%)	34 (34.0%)		
Neonatal jaundice (ABO)	0 (0.0%)	10 (10.0%)		
Pneumonia	63 (63.0%)	0 (0.0%)		
Severe TTN	37 (37.0%)	16 (16.0%)		
Respiratory support				
No	0 (0.0%)	44 (44.0%)	χ^2^ = 146.563	<0.001 *
CPAP	45 (45.0%)	19 (19.0%)		
MV	55 (55.0%)	0 (0.0%)		
Nasal cannula	0 (0.0%)	37 (37.0%)		
Outcome				
Discharged	90 (90.0%)	100 (100.0%)	χ^2^ = 10.526 *	0.001 *
Died	10 (10.0%)	0 (0.0)		
AKI development Yes No	52 (52.0%) 48 (48%)	-	-	-
Antibiotics intake Yes No	51 (51.0%) 49 (49.0%)	-	-	-

U: Mann–Whitney test, t: Student *t*-test, χ^2^: Chi-square test, * *p*: *p*-value for comparing between the two studied groups is statistically significant at *p* ≤ 0.05.

**Table 2:** Comparison between the two studied groups in terms of different biochemical parameters.

	Case (*n* = 100)	Control (*n* = 100)	Test of Sig	*p*
PH Mean ± SD.	7.35 ± 0.11	4.07 ± 3.63	1645.50	<0.001 *
HCO3 (mmol/L) Mean ± SD.	25.79 ± 4.04	11.27 ± 10.12	657.00	<0.001 *
Na (mmol/L)				
Mean ± SD.	137.49 ± 3.73	138.70 ± 2.47	t = 2.707	0.007 *
K (mmol/L)				
Mean ± SD.	4.37 ± 0.40	4.43 ± 0.38	t = 1.065	0.288
Ca (mg/dL)				
Mean ± SD.	8.66 ± 0.50	8.73 ± 0.52	t = 1.049	0.295
RBS (mg/dL)				
Mean ± SD.	69.70 ± 9.43	71.04 ± 9.31	t = 1.011	0.313
HB G/dL				
Mean ± SD.	14.98 ± 1.74	15.47 ± 1.15	t = 2.373	0.019 *
Platelets				
Mean ± SD.	242.43 ± 35.92	230.78 ± 18.39	t = 2.887	0.004 *
TLC				
Mean ± SD.	17.44 ± 4.97	11.53 ± 1.61	t = 11.30	<0.001 *
ALT (U/L)				
Mean ± SD.	31.60 ± 3.05	32.20 ± 3.49	t = 1.295	0.197
AST (U/L)				
Mean ± SD.	33.51 ± 3.35	33.52 ± 3.10	t = 0.022	0.983
Urea (mg/dL)				
Mean ± SD.	26.51 ± 3.87	26.32 ± 3.52	t = 0.363	0.717
Creatinine (mg/dL)				
Mean ± SD.	0.75 ± 0.11	0.73 ± 0.11	t = 1.260	0.209
Lactate (mg/dL)				
Median (IQR)	1.20 (0.89)	0.90(0.57)	U = 3199	<0.001 *
Cyst C (mg/L)				
Median (IQR)	12.15 (5.9)	6.0(3)	U = 949	<0.001 *
CRP (mg/dL) Median (IQR)	36.0 (60.0)	-	-	-

U: Mann–Whitney test, t: Student *t*-test * *p*: *p*-value for comparing between the two studied groups is statistically significant at *p* ≤ 0.05.

**Table 3:** Comparison between the two studied groups according to TNF-α (−238, rs361525).

	Case (*n* = 100)	Control (*n* = 100)	Test of Sig.	*p*
TNF-α (−238, rs361525)				
GG	50 (50.0%)	67 (67.0%)	χ^2^ = 8.779 *	0.012 *
GA	36 (36.0%)	29 (29.0%)		
AA	14 (14.0%)	4 (4.0%)		
^HW^p_0_	0.084	0.702		
Allele				
G	136 (68.0%)	163 (81.5%)	χ^2^ = 9.656 *	0.002 *
A	64 (32.0%)	37 (18.5%)		

χ^2^: Chi square test; *p*: *p* value for comparing the two studied groups. ^HW^p_0_: *p* value for Chi square for goodness of fit for Hardy-Weinberg equilibrium (If *p* < 0.05—not consistent with HWE) *: Statistically significant at *p* ≤ 0.05.

**Table 4:** Cystatin C levels in different TNF-α (−238, rs361525) genotypes among critically neonates with sepsis.

	TNF-α (−238, rs361525)			K	*p* Value	Sig. Bet. Groups	
	GG (*n* = 55)	GA (*n* = 31)	AA (*n* = 14)				
Cystatin C (mg/L) Median (IQR)	7.9 (5.2)	12.39 (2.5)	13.8 (2.7)	31.8	<0.001 **	GG-GA	0.001 **
						GG-AA	0.001 **
						GA-AA	0.32

K: Kruskal–Wallis test, *p*: *p*-value for comparing between the two studied groups, **: highly significant at *p* ≤ 0.01.

**Table 5:** Comparison between creatinine and urea levels within the case group.

	Case (*n* = 100)			Test of Sig	*p*	Wilcoxon Signed Ranks Test
	1st	2nd	3rd			
Creatinine (mg/dL)						
Median (IQR)	1.10 (0.5)	1.50 (0.8)	1.57 (1.8)	χ^2^ = 69.9	<0.001 *	*p* ≤ 0.001 ^a^
Urea (mg/dL)						
Median (IQR)	45.0 (30)	92.0 (55.3)	92.0 (55.3)	χ^2^ = 70.4	<0.001 *	*p* ≤ 0.001 ^a^

Friedman’s Anova Test, * *p*: *p*-value for comparing the different readings is highly statistically significant at *p* ≤ 0.01 compared to 1st readings. ᵃ Wilcoxon Signed-Ranks Test.

**Table 6:** TNF-α (−238, rs361525) and Cyst C in AKI and non-AKI cases.

	AKI (*n* = 52)	Non-AKI (*n* = 48)	Test of Sig.	*p*
TNF-α (−238, rs361525)				
GG	15 (28.8%)	35 (72.9%)	χ^2^ = 24.657	<0.001 *
GA	23 (44.2%)	13 (27.1%)		
AA	14 (26.9%)	0 (0.0%)		
^HW^p_0_	0.407	0.278		
Allele				
G	53 (51.0%)	83 (86.5%)	χ^2^ = 28.906 *	<0.001 *
A	51 (49.0%)	13 (3.5%)		
**Cyst C**				
Median (IQR)	13.05(2.2)	7.90(4.3)	U = 380.50 *	<0.001 *

χ^2^: Chi square test, U: Mann–Whitney test, *p*: *p*-value for comparing the two studied groups. ^HW^p_0_: *p* value for Chi square for goodness of fit for Hardy-Weinberg equilibrium (If *p* < 0.05—not consistent with HWE). *: Statistically significant at *p* ≤ 0.05.

**Table 7:** Prognostic performance of Cyst C in predicting AKI (*n* = 52) vs. non-AKI (*n* = 48).

	AUC	*p*	95% CI	Cut Off	Sensitivity	Specificity	PPV	NPV
Cyst C	0.848	<0.001 *	0.767–0.928	>9.4	88.46	75.0	79.3	85.7

AUC: Area under the curve, *p*-value: Probability value, CI: Confidence intervals. NPV: Negative predictive value PPV: Positive predictive value *: Statistically significant at *p* ≤ 0.05.

**Table 8:** Agreement (sensitivity, specificity, and accuracy) for TNF-α (−238, rs361525) and Cyst C.

	Non-AKI (*n* = 48)	AKI (*n* = 52)	Sensitivity	Specificity	PPV	NPV	Accuracy
GA + AA	13 (27.1%)	37 (71.2%)	71.15	72.92	74.0	70.0	72.0
GA + AA & Cyst C (>9.4 mg/L)	8 (16.7%)	35 (67.3%)	67.31	83.33	81.40	70.18	75.0

PPV: Positive predictive value; NPV: Negative predictive value.

**Table 9:** Univariate regression analysis to detect the association between AKI with TNF-α (−238, rs361525) and Cyst C in the cases group (*n* = 100).

	AKI (*n* = 52)	Non-AKI (*n* = 48)	*p*	OR (LL–UL 95% CI)
TNF-α (−238, rs361525)				
GG^®^	15 (28.8%)	35 (72.9%)		1.000
GA	23 (44.2%)	13 (27.1%)	0.002 **	4.128 (1.661–10.257)
AA	14 (26.9%)	0 (0.0%)	NA	NA
^HW^p	0.407	0.278		
GA + AA	37 (71.2%)	13 (27.1%)	<0.001 **	6.641 (2.769–15.927)
Allele				
G	53 (51.0%)	83 (86.5%)		1.000
A	51 (49.0%)	13 (3.5%)	<0.001 **	6.1437 (3.052 –12.368)
Cyst C (mg/L)				
Median (IQR)	13.05 (2.2)	7.90 (4.3)	<0.001 **	1.759(1.439–2.150)

OR: Odds ratio. ^®^: reference group. CI: Confidence interval, LL: Lower limit, UL: Upper limit, *p*: *p*-value for univariate regression analysis, **: Highly statistically significant at *p* ≤ 0.01.

**Table 10:** The association between different parameters and AKI development in septic critically ill patients.

	Univariate		^#^ Multivariate	
	*p*	OR (LL–UL 95% CI)	*p*	OR (LL–UL 95% CI)
Age (months)	0.163	1.006 (0.998–1.014)		
Down score	0.459	1.222 (0.719–2.078)		
Platelets	0.476	1.004 (0.993–1.015)		
TLC	0.448	1.032 (0.952–1.118)		
Urea (mg/dL)	0.057	0.900 (0.807–1.003)		
Creatinine (mg/dL)	0.246	7.927 (0.240–261.998)		
Cyst C	<0.001 *	1.759 (1.439–2.150)	<0.001 *	1.627 (1.299–2.038)
Lactate	0.002 *	0.308 (0.146–0.647)	0.586	0.763 (0.288–2.022)
CRP (mg/dL)	0.274	1.007 (0.994–1.020)		
TNF-α (−238, rs361525) (GA + AA)	<0.001 *	6.641 (2.769–15.927)	0.234	1.968 (0.645–6.004)

OR: Odds ratio; CI: Confidence interval; LL: Lower limit; UL: Upper limit. ^#^: All variables with *p* < 0.05 were included in the multivariate analysis; *: Statistically significant at *p* ≤ 0.05.

## Data Availability

Data supporting reported results will be made available from the corresponding author upon reasonable request.
